# Multiscale oxygen transfer enhancement in high‑density *Komagataella phaffii* fermentation: dynamic pressure control and CFD‑guided industrial scale‑up

**DOI:** 10.1186/s40643-026-01155-9

**Published:** 2026-10-28

**Authors:** Qiang Duan, Fengchao Wang, Qinran Ding, Bo Xiong, Baocai Zhang, Huan Yu, Junqi Zhang, Yaru Chen, Chao Li, Qitian Huang, Hao Song

**Affiliations:** 1 State Key Laboratory of Synthetic Biology, Tianjin University, Tianjin, 300350 China; 2 Frontiers Science Center for Synthetic Biology (Ministry of Education), Tianjin University, Tianjin, 300350 China; 3 School of Synthetic Biology and Biomanufacturing, Tianjin University, Tianjin, 300350 China; 4 Weifang KDN Biotech Co., Ltd., Weifang, 261500 Shandong China; 5 Institute of Synthetic Biology, College of Life and Health Sciences, Northeastern University, Shenyang, 110169 China

**Keywords:** *Komagataella phaffii*, Oxygen transfer, Dynamic pressure control, Computational fluid dynamics, Scale-up, Impeller system optimization

## Abstract

Oxygen transfer limitation is a key bottleneck in high-cell-density *Komagataella phaffii* fermentation for recombinant protein production. Conventional approaches based on increasing agitation and aeration may result in high shear stress, foaming, and increased energy demand. In this study, a multiscale oxygen-transfer enhancement strategy integrating dynamic headspace-pressure regulation with computational fluid dynamics (CFD)-guided bioreactor optimization was investigated. At the 5 L scale, response surface methodology demonstrated the combined effects of agitation, aeration, and headspace pressure on the volumetric oxygen-transfer coefficient ($k_{L} a$). A two-stage dynamic pressure (TSP) strategy was subsequently evaluated under fermentation conditions. Compared with the conventional-pressure condition, TSP increased the final dry cell weight (DCW) by 11.2% and the maximum xylanase activity by 19.8%, reaching 120.25 ± 1.04 g/L and 213.23 ± 2.10 KU/mL, respectively. The strategy was further implemented in a 1000 L bioreactor, where the peak oxygen uptake rate reached 185.23 mmol/L/h, with a final DCW of 124.08 ± 2.47 g/L and xylanase activity of 225.07 ± 3.64 KU/mL. At the 30 m^3^ scale, CFD analysis identified scale-dependent hydrodynamic limitations in the original agitation system and guided a combined redesign from 0.7 m Rushton turbines to a 0.8 m CD-6/3KSX hybrid impeller system. Under identical simulation conditions, the redesigned configuration increased the CFD-predicted average $k_{L} a$ by 46.4% and reduced the predicted mixing time by 43.3%. Following implementation of the redesigned system, the final DCW and xylanase activity reached 134.01 ± 2.98 g/L and 242.02 ± 4.07 KU/mL, respectively. These results demonstrate a case-based multiscale approach combining process-level oxygen-transfer regulation with reactor-level hydrodynamic optimization for the investigated recombinant *K. phaffii* fermentation process.

**Graphical abstract:**

**Figure:**
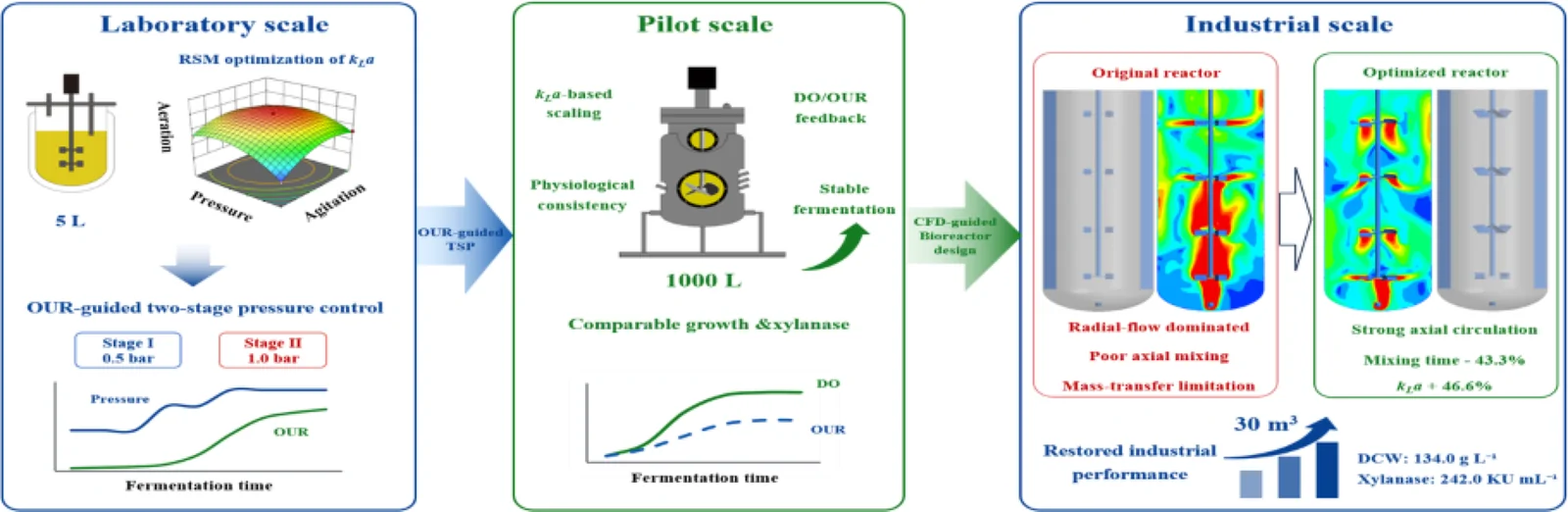

**Supplementary Information:**

The online version contains supplementary material available at https://doi.org/10.1186/s40643-026-01155-9.

## Introduction

The methylotrophic yeast *Komagataella phaffii* (previously described as *Pichia pastoris*) garnered extensive interests as a predominant platform for industrial production of recombinant proteins, owing to its strong inducible AOX1 promoter for high-level protein expression, ability of post-translational modifications, and capacity of high-cell-density fermentation (Cereghino and Cregg 2000; Macauley-Patrick et al. 2005). In high-cell-density fermentation, the oxygen uptake rate (OUR) rises sharply as biomass accumulates, which may lead to vigorous methanol metabolism (Jahic et al. 2006). However, the inherently low solubility of oxygen in aqueous culture frequently leads to rapid depletion of dissolved oxygen (DO), which could generate severe oxygen transfer limitation that is deemed to a critical bottleneck in the *K. phaffii* fermentation process (Garcia-Ochoa and Gomez 2009). Severe oxygen limitation may impair methanol metabolism and alter by-product formation, thereby adversely affecting cell growth and recombinant protein production (Inan and Meagher 2001; Charoenrat et al. 2005; Khatri and Hoffmann 2006).

Conventional strategies to alleviate the DO limitation primarily rely on increasing the agitation speed and aeration rate, or employing oxygen-enriched air (Garcia-Ochoa and Gomez 2009). Nevertheless, these approaches have intrinsic limitations: excessive agitation produces high shear stress that may compromise cell viability (Garcia-Ochoa et al. 2010; García-Ochoa et al. 2013); high aeration rates frequently result in impeller flooding as well as excessive foam formation; and oxygen enrichment is often constrained by high operational costs (Liu et al. 2016). In contrast, adjusting the bioreactor headspace pressure provides an alternative engineering approach for alleviating DO limitation. According to the Henry’s Law, elevating the partial pressure of oxygen directly increases its saturation concentration ($\:{C}^{*}$) in the liquid phase, thereby enhancing the driving force ($\:{C}^{*}-{C}_{l}$) for mass transfer without intensifying the hydrodynamic shear (Pinheiro et al. 2003; Lopes et al. 2013). Previous studies have demonstrated that elevated air pressure can enhance oxygen availability and support high-cell-density *K. phaffii* cultivation at laboratory and larger scales (Knoll et al. 2005; Lopes et al. 2013; Liu et al. 2016). However, pressure in these studies was mainly applied as a predefined operating condition or scale-up measure, whereas its use as a stage-dependent operating variable in response to changes in cellular oxygen demand remains relatively underexplored.

Furthermore, the scale-up of aerobic bioprocesses from laboratory to industrial scales represents a formidable challenge. Empirical criteria such as specific power input per unit volume (* P/V*) or volumetric oxygen transfer coefficient ($\:{k}_{L}a$), cannot frequently ensure stable performance of large-scale bioreactors, owing to the nonlinear changes in hydrodynamics and emergence of spatial heterogeneities in large-scale bioreactors (Garcia-Ochoa and Gomez 2009; Xu et al. 2017). At the scales of tens of cubic meters’ bioreactors, prolonged mixing times could lead to significant gradients in the DO and substrate concentrations, which often create suboptimal microenvironments that reduce cell viability and productivity (Castan and Enfors 2002; Arulrajah et al. 2025). In the industrial-scale bioreactors, manipulating operational parameters alone is often insufficient to overcome intrinsic hardware constraints. Computational fluid dynamics (CFD) has been proven to be a reliable diagnostic tool for visualizing multiphase flow fields and quantifying mixing inefficiencies, supporting the rational design and optimization of bioreactor hardware for successful scale-up (Gogate et al. 2000; Gu et al. 2026).

Therefore, this study aimed to investigate a multiscale oxygen-transfer enhancement strategy integrating dynamic pressure regulation with reactor engineering for high-density *K. phaffii* fermentation. At the 5 L scale, response surface methodology (RSM) was first used to characterize the effects of agitation, aeration, and headspace pressure on oxygen transfer, followed by evaluation of a two-stage dynamic pressure (TSP) strategy. The strategy was subsequently implemented at the 1000 L pilot scale using engineering scale-up criteria, while CFD was further employed at the 30 m^3^ industrial scale to characterize hydrodynamic limitations and guide redesign of the agitation system. This study thus integrates process-level oxygen-transfer regulation with reactor-level hydrodynamic optimization across fermentation scales.

## Materials and methods

### Microbial strain, fermentation medium, and cultivation conditions

The methanol-utilization plus (Mut^+^) strain *K. phaffii* was used for the recombinant expression of xylanase under the control of the inducible alcohol oxidase 1 (AOX1) promoter. The fermentation culture medium contained components (per liter): glucose, 22 g; 85% phosphoric acid (H₃PO₄), 16.5 mL; magnesium sulfate heptahydrate (MgSO₄·7 H₂O), 13.5 g; calcium sulfate (CaSO₄), 0.56 g; potassium sulfate (K₂SO₄), 10.4 g; potassium hydroxide (KOH), 2.3 g; PTM_1_ trace salts solution, 3.6 mL; and antifoam agent, 1.5 mL.

The fermentation process was strictly controlled at 30 °C, and the pH was maintained at 5.0 ± 0.1 using 23% (v/v) ammonium hydroxide. The initial working volumes were 3.5 L and 700 L for the 5 L and 1000 L bioreactors, respectively. Seed cultures in the exponential growth phase were used for inoculation at 10% (v/v). The entire procedure consisted of three sequential phases:

Batch phase (Phase I): Cells were cultivated in the initial glucose-based medium until glucose exhaustion, which was indicated by a sharp DO spike.

Glucose fed-batch phase (Phase II): A glucose-limited feeding strategy was implemented by supplying a 50% (w/w) glucose solution supplemented with PTM_1_ trace salts (12 mL/L). The feeding followed a preset exponential profile to sustain near-exponential growth. Glucose feeding was terminated when the cell dry weight (DCW) reached approximately 40 g/L, followed by a 0.5-hour starvation period to ensure complete depletion of the residual glucose.

Methanol induction phase (Phase III): Induction was initiated by commencing a continuous feed of pure methanol containing PTM_1_ trace salts (12 mL/L). The methanol-feeding profile was selected based on previous process-development experience and reported feeding strategies for Mut^+^ *K. phaffii*, in which methanol supply is progressively increased following adaptation to methanol to sustain induction while avoiding excessive methanol accumulation (Maghsoudi et al. 2012; Liu et al. 2016). During the transition period, the methanol feed rate was therefore gradually increased from 2.0 to 6.2 mL/h/L of initial broth volume. In the mid-induction phase, as biomass and methanol-utilization capacity increased, the feed rate was further raised to approximately 9.7 mL/h/L and maintained until the end of fermentation. 

### Experimental design of $\:{\boldsymbol{k}}_{\boldsymbol{L}}\boldsymbol{a}$ optimization and pressure strategy evaluation

#### Response surface methodology (RSM) for $\:{k}_{L}a$ optimization

The volumetric oxygen transfer coefficient ($\:{k}_{L}a$) is the key parameter governing oxygen supply in aerobic bioreactors (Stanbury et al. 2016; Garcia-Ochoa et al. 2020). To systematically investigate and optimize the effects of key engineering parameters on oxygen transfer, the response surface methodology (RSM) was thus employed. A Box-Behnken Design (BBD) with three factors at three levels was implemented using the Design-Expert software (Table 1). The independent variables were: agitation speed (A: 500–900 rpm), aeration rate (B: 1.5–2.5 vvm (volume of gas per volume of liquid per minute), and bioreactor headspace pressure (C: 0.5–1.5 bar). Unless otherwise stated, all headspace pressure values reported in this study refer to gauge pressure. The response variable was the $\:{k}_{L}a$ value (h⁻¹). To distinguish the physical effects of these parameters from biological variability, all RSM experiments were conducted in a 5 L bioreactor filled with pure water at 30 °C, and $\:{k}_{L}a$ was determined via the dynamic gassing-out method (Tribe et al. 1995). The water-based $\:{k}_{L}a$ measurements were used as a standardized engineering basis for comparing the relative effects of agitation, aeration, and headspace pressure, rather than as direct estimates of the absolute $\:{k}_{L}a$ under high-cell-density fermentation conditions. A total of 15 experimental runs, including three center-point replicates, were performed in random order (Table 2). The experimental data were fitted to a second-order polynomial equation, and analysis of variance (ANOVA) was employed to assess the statistical significance of the model. The stationary point of the fitted response surface was calculated to identify the model-predicted maximum within the experimental design space.

**Table 1:** Levels and codes of variables for the Box-Behnken design

Independent variables	Coded variables	Coded levels		
		− 1	0	1
Agitation (rpm)	A	500	700	900
Aeration (vvm)	B	1.5	2.0	2.5
Fermentor pressure (bar)	C	0.5	1.0	1.5

**Table 2:** Experimental design and the response values of the fermentor

Experiment number	Agitation (rpm)	Aeration (vvm)	Fermentor pressure (bar)	$k_{L} a$ (h^− 1^)
1	500 (-1)	1.5 (− 1)	1.0 (0)	76.5
2	500 (-1)	2.5 (1)	1.0 (0)	89.4
3	900 (1)	1.5 (− 1)	1.0 (0)	120.8
4	900 (1)	2.5 (1)	1.0 (0)	154.4
5	500 (-1)	2.0 (0)	0.5 (− 1)	77.1
6	500 (-1)	2.0 (0)	1.5 (1)	81.6
7	900 (1)	2.0 (0)	0.5 (− 1)	101.7
8	900 (1)	2.0 (0)	1.5 (1)	170.5
9	700 (0)	1.5 (− 1)	0.5 (− 1)	65.7
10	700 (0)	1.5 (− 1)	1.5 (1)	124.4
11	700 (0)	2.5 (1)	0.5 (− 1)	129.3
12	700 (0)	2.5 (1)	1.5 (1)	132.9
13	700 (0)	2.0 (0)	1.0 (0)	189.6
14	700 (0)	2.0 (0)	1.0 (0)	196.7
15	700 (0)	2.0 (0)	1.0 (0)	192.4

#### Design of headspace pressure control strategies

In industrial fermentation processes, maintaining a slightly positive headspace pressure is a common practice to avoid microbial contamination (Barone et al. 2023). However, the appropriate pressure range may vary depending on the factors such as fermentation type, strain characteristics, and production scale (Knoll et al. 2005). Based on the RSM results, the role of headspace pressure was further evaluated under actual fermentation conditions using three distinct pressure-control strategies during *K. phaffii* fermentation. To isolate the effect of headspace-pressure regulation, the Control, HP, and TSP fermentations were initiated using the same strain, inoculation procedure, and initial cultivation conditions, and were subsequently operated using identical feeding, agitation, and aeration profiles. In all three groups, agitation and aeration followed the same predefined time-dependent profiles throughout fermentation. Agitation was increased stepwise from 300 to 400, 500, 600, and 700 rpm, while aeration was increased from 1.0 to 1.5 and subsequently to 2.0 vvm. The timing and magnitude of these changes were identical among the three treatments; the headspace-pressure profile was the only intentionally varied oxygen-transfer operating variable.

The three headspace pressure control strategies that were applied in this study included:

1. Constant conventional pressure (Control): The headspace pressure was maintained constant at 0.5 bar throughout the fermentation process, simulating the typical industrial slight overpressure operation.
2. Constant conventional pressure (Control): The headspace pressure was maintained constant at 0.5 bar throughout the fermentation process, simulating the typical industrial slight overpressure operation.
3. Two-stage dynamic pressure (TSP): During the cell growth phase and the early methanol induction stage, the conventional pressure of 0.5 bar was maintained. Upon entering the mid-to-late induction phase, where oxygen consumption rose with increased methanol metabolism, the headspace pressure was gradually increased to 1.0 bar. This strategy aimed to investigate a pressure-control mode that dynamically responded to cellular oxygen demand while avoiding unnecessary prolonged operation at elevated pressure.

### Analytical methods

#### Measurement of $\:{k}_{L}a$

The determination of the volumetric oxygen mass transfer coefficient ($\:{k}_{L}a$) in bioreactors is essential in evaluating aeration efficiency and analyzing the influence of operational parameters on the DO supply (Garcia-Ochoa and Gomez 2009; Stanbury et al. 2016). The DO mass balance in a homogeneous liquid phase system can be expressed as:

1 $$ {\frac{{dC}}{{dt}} = OTR - OUR} $$

where $\:\frac{dC}{dt}$ is the oxygen accumulation rate in the liquid phase, * OTR* is the oxygen transfer rate, and * OUR* is the oxygen uptake rate. Based on the two-film theory and the Henry’s law (Sander 2023), * OTR* can be described as:

2 $$ \begin{array}{*{20}c} {OTR = k_{L} a \cdot \left( {C^{*} - C_{l} } \right)} \\ \end{array} $$

where $\:{C}^{*}$ is the DO saturation concentration in equilibrium with the gas phase, and $\:{C}_{l}$ is the actual DO concentration in the liquid bulk. When required for oxygen-solubility calculations, gauge pressure was converted to absolute pressure according to $\:{P}_{abs}={P}_{g}+{P}_{atm}$.

In this study, $\:{k}_{L}a$ was determined in a 5 L stirred bioreactor at 30 °C using the dynamic gassing-out method under cell-free conditions. Because no respiring microorganisms were present during the measurement, * OUR* was assumed to be negligible, and Eq. (1) was simplified to:

3 $$ {\frac{{dC}}{{dt}} = k_{L} a \cdot \left( {C^{*} - C_{l} } \right)} $$

For each operating condition, the liquid phase was first deoxygenated by nitrogen sparging until the DO level decreased below approximately 10% air saturation. Nitrogen was then rapidly replaced with air while agitation speed, aeration rate, and headspace pressure were maintained at their predefined values. The subsequent DO recovery was continuously monitored using a rapid-response polarographic oxygen electrode (InPro 6860i, Mettler Toledo). DO data were recorded at 1-s intervals, and the portion of the DO recovery curve between 20% and 80% air saturation was used for linear regression. This interval provided sufficient data points for regression under all tested operating conditions.

Integration of Eq. (3) gives:

4 $$ {\ln \left( {\frac{{C^{*} - C_{L} }}{{C^{*} - C_{{L,0}} }}} \right) = - k_{L} a \cdot t} $$

where $\:{C}_{L,0}$ is the dissolved oxygen concentration at the beginning of the selected fitting interval. The $\:{k}_{L}a$ value was obtained from the absolute slope of the linear regression. Only regressions with R^2^ $\:\ge\:$ 0.98 were accepted. Each operating condition was measured in triplicate, and the results were expressed as mean ± standard deviation.

#### Determination of dry cell weight (DCW) and metabolic parameters

Cell concentration was determined spectrophotometrically by measuring the optical density at 600 nm (OD_600_). Fermentation samples were appropriately diluted to maintain absorbance within the linear range of the spectrophotometer. A strain-specific OD_600_–DCW calibration was established using fermentation samples with different cell densities. Gravimetric DCW was determined after centrifugation, washing, and drying to constant weight. The calibration showed good linearity (Fig. S1), and the resulting relationship was used for subsequent DCW determination.

The oxygen mole fractions in the inlet and exhaust gases were continuously monitored using a mass spectrometer (SHP8400PMS, Shanghai Sunny Hengping Scientific Instrument, China), and the oxygen uptake rate (OUR) was calculated from the gas-phase oxygen balance according to Eq. (5). Volumetric gas-flow rates were corrected for pressure, temperature, and water-vapor content and converted to dry-gas molar flow rates, with the same correction procedure applied at the 5 L, 1000 L, and 30 m^3^ scales.

The oxygen molar balance was expressed as:

5 $$ {OUR = \frac{{F_{{in}} y_{{O_{2} ,in}} - F_{{out}} y_{{O_{2} ,out}} }}{{V_{L} }}} $$

where $\:{F}_{in}$ and $\:{F}_{out}$ are the corrected molar flow rates of the inlet and exhaust gases, respectively; $\:{y}_{{O}_{2},in}$ and $\:{y}_{{O}_{2},out}$ are the corresponding oxygen mole fractions; and $\:{V}_{L}$ is the instantaneous broth volume. 

#### Xylanase activity assay

Xylanase activity was determined using the 3,5-dinitrosalicylic acid (DNS) reducing sugar method, adapted from Bailey et al. (Bailey et al. 1992). For the assay, 0.5 mL of appropriately diluted fermentation supernatant was mixed with 0.5 mL of 1% (w/v) oat spelt xylan in 50 mM citrate buffer (pH 5.0) and incubated at 50 °C for 30 min. The reaction was terminated by adding 1.5 mL of DNS reagent, followed by heating in a boiling water bath for 10 min. After cooling to room temperature, the absorbance was measured at 540 nm. A D-xylose standard curve covering 0.1–1.0 mg/mL was used to quantify the released reducing sugars, and a blank in which DNS reagent was added before the enzyme sample was used for background correction.

One unit of xylanase activity (U) was defined as the amount of enzyme required to release 1 µmol of xylose equivalents per minute under the assay conditions described above. Xylanase activity was expressed as kilo units per milliliter of fermentation supernatant (KU/mL). For each fermentation condition, three independent fermentation batches were performed as biological replicates, and each xylanase assay was conducted in technical triplicate. The reported values are presented as mean ± standard deviation of the three biological replicates.

To compare xylanase production among the different pressure-control strategies and fermentation scales, the overall volumetric xylanase productivity ($\:{Q}_{P}$) was calculated as:

6 $$ {Q_{P} = \frac{{P_{f} }}{{t_{f} }}} $$

where $\:{P}_{f}$ is the final xylanase activity (KU/mL) and $\:{t}_{f}$ is the corresponding fermentation time (h). The overall biomass-specific productivity ($\:{q}_{P}$) was calculated as:

7 $$ {q_{P} = 1000\frac{{Q_{P} }}{{X_{f} }}} $$

where $\:{X}_{f}$ is the final dry cell weight (g/L). $\:{Q}_{P}$ and $\:{q}_{P}$ were expressed as KU/mL^/^h and KU/g_DCW_/h, respectively.

### Pilot-scale validation and dynamic control strategy

To translate the laboratory-scale oxygen-transfer strategy to the 1000 L pilot-scale bioreactor, feasible agitation and aeration operating ranges were first defined based on engineering scale-up considerations, followed by DO-responsive adjustment of agitation, aeration, and headspace pressure during fermentation.

The agitation range was scaled using approximately constant specific mechanical power input (* P/V*) as the engineering criterion. For a stirred tank, the mechanical power input can be expressed as:

8 $$ {P = N_{P} \rho N^{3} D^{5} } $$

where * P* is the mechanical power input (W), $\:{N}_{P}$ is the impeller power number, $\:\rho\:$ is the liquid density (kg/m^3^), * N* is the agitation rate (s^− 1^), and * D* is the impeller diameter (m). Assuming comparable liquid properties and power numbers for the 5 L and 1000 L bioreactors, the agitation-speed scale factor under approximately constant * P/V* can be expressed as:

9 $$ {\frac{{N_{2} }}{{N_{1} }} = \left[ {\frac{{V_{2} }}{{V_{1} }}\left( {\frac{{D_{1} }}{{D_{2} }}} \right)^{5} } \right]^{{1/3}} } $$

where $\:{V}_{1}$ and $\:{V}_{2}$ are the working volumes of the 5 L and 1000 L bioreactors, respectively. The corresponding working volumes were 3.5 and 700 L, and the impeller diameters were approximately 68 and 400 mm. These values yielded an agitation-speed scale factor of approximately 0.305, translating the 5 L agitation range of 300–700 rpm to approximately 93–217 rpm at the 1000 L scale. Based on the same assumptions, the calculated specific-power ratio between the implemented 1000 L and 5 L conditions was approximately 1.05. Because shaft torque and actual gassed mechanical power were not measured directly, * P/V* was used as an engineering scale-up approximation rather than as an experimentally determined gassed-power value.

Aeration was not scaled by maintaining the same vvm because direct volumetric scaling could increase the superficial gas velocity and the risks of excessive foaming, gas entrainment, and impeller flooding in the larger vessel. A feasible aeration range of 0.6–1.2 vvm, approximately 60% of the corresponding 5 L values, was therefore adopted for the 1000 L bioreactor. This range was selected based on pilot-scale hydrodynamic operability and oxygen-transfer demand rather than derived directly from the 5 L RSM design (Xu et al. 2017).

During the 1000 L fermentation, agitation was increased stepwise from 93 to 124, 155, 186, and finally 217 rpm, while aeration was increased from 0.6 to 0.9 and subsequently to 1.2 vvm as DO declined. During methanol induction, headspace pressure was increased when DO continued to decrease after agitation and aeration had approached their predefined upper limits. At approximately 52 h, when DO decreased to about 25%, the headspace pressure was increased from 0.5 to 0.75 bar; when DO again approached 25% at approximately 58 h, the pressure was further increased to 1.0 bar. Thus, a DO level of approximately 25% served as the direct operational trigger for pressure elevation. OUR was monitored simultaneously as an indicator of cellular oxygen demand and process state, but was not used as the direct pressure-control trigger. The switching times therefore reflected the DO response of the fermentation rather than preset time points.

### CFD modeling

At the 30 m^3^ scale, adjustment of agitation and aeration within the feasible operating range did not consistently maintain the expected fermentation performance. Computational fluid dynamics (CFD) was therefore employed to characterize the gas–liquid hydrodynamics and oxygen-transfer characteristics of the industrial fermenter and to identify potential limitations associated with the agitation system (Panunzi et al. 2022).

A three-dimensional model was constructed according to the actual geometric parameters of the industrial fermenter listed in Table 3, and simulations were performed using ANSYS Fluent (ANSYS Inc., Canonsburg, PA, USA). Polyhedral meshes were generated using Fluent Meshing, with local refinement around the impeller blades, gas inlet, and baffles. Two agitation configurations were investigated: the original four-stage Rushton turbine system with an impeller diameter of 0.7 m (Fig. S2a) and the redesigned CD-6/3KSX hybrid system with an impeller diameter of 0.8 m (Fig. S2b). The final meshes contained approximately 1.25 million and 1.30 million polyhedral cells for the original and redesigned configurations, respectively. Details of the mesh-independence analysis are provided in the Supplementary Information (Fig. S3).

**Table 3:** Key geometric specifications of the 30 m^3^ fermenter before and after optimization of the impeller system

Parameter		Before optimization	After optimization
*General specifications*			
Nominal volume (m^3^)		30	30
Internal diameter (mm)		2300	2300
Shaft diameter (mm)		100	100
Working volume (m^3^)		24	24
Gas inlet pipe diameter (mm)		120	120
*Impeller configuration (Top → Bottom)*			
Impeller I	Type	Rushton Turbine (6-flat blade)	3KSX (4-wide blade)
	Diameter (mm)	700	800
	Spacing below (mm)	1600	1600
Impeller II	Type	Rushton Turbine (6-flat blade)	3KSX (4-wide blade)
	Diameter (mm)	700	800
	Spacing below (mm)	1600	1600
Impeller III	Type	Rushton Turbine (6-flat blade)	3KSX (4-wide blade)
	Diameter (mm)	700	800
	Spacing below (mm)	1300	1300
Impeller IV	Type	Rushton Turbine (6-flat blade)	CD-6 (6-half pipe turbine)
	Diameter (mm)	700	800
	Spacing to bottom (mm)	975	975

Gas–liquid flow was described using an Eulerian–Eulerian two-fluid framework. Turbulence in the continuous liquid phase was modeled using the standard $\:k-\epsilon\:$ model. Air was defined as the dispersed gas phase, whereas water was used as the reference liquid phase, with a density of 1000 kg/m^3^ and a dynamic viscosity of 0.001 Pa·s. Interphase momentum exchange was described using the Universal drag model, whereas lift, virtual-mass, and other secondary interphase forces were neglected. Bubble-size evolution was resolved using a discrete Population Balance Model (PBM). The bubble population was divided into 10 discrete size bins with diameters ranging from approximately 1 to 30 mm and a geometric size ratio of 1.65. Bubble coalescence was described using the Prince-Blanch aggregation model (Prince and Blanch 1990), bubble breakage was described using the Luo model (Luo and Svendsen 1996), and the daughter-bubble size distribution was represented using the Laakkonen probability density function with the Hagesaether formulation.

The local liquid-side oxygen mass-transfer coefficient was calculated using the small-eddy model:

10 $$ {k_{L} = 0.4D_{L} ^{{0.5}} \left( {\frac{\varepsilon }{{v_{L} }}} \right)^{{0.25}} } $$

where $\:{D}_{L}$ is the molecular diffusivity of oxygen in the liquid phase, $\:\epsilon\:$ is the local turbulent energy dissipation rate, and $\:{v}_{L}$ is the liquid-phase kinematic viscosity. The local gas–liquid interfacial area was calculated as:

11 $$ {a = \frac{{6\alpha _{g} }}{{d_{b} }}} $$

where $\:{\alpha\:}_{g}$ is the local gas volume fraction and $\:{d}_{b}$ is the local representative bubble diameter obtained from the PBM. The local volumetric oxygen-transfer coefficient was subsequently calculated as:

12 $$ {k_{L} a = k_{L} \cdot a} $$

and the overall $\:{k}_{L}a$ of the fermenter was obtained by volume averaging the local values over the liquid domain.

The gas inlet was specified as a velocity-inlet boundary condition corresponding to an aeration rate of 1.18 vvm, the top gas outlet as a pressure outlet, and all solid walls as no-slip boundaries. Impeller rotation at 155 rpm was represented using the Multiple Reference Frame (MRF) approach, and the flow field was solved under steady-state conditions. Pressure–velocity coupling was performed using the Phase-Coupled SIMPLE algorithm, with the Least-Squares Cell-Based method for gradients and PRESTO! for pressure interpolation. First-order upwind discretization was applied to momentum, phase volume fraction, turbulent kinetic energy, turbulent dissipation rate, and the transported PBM equations. Convergence was considered achieved when the scaled residuals decreased below 10^− 4^ and the overall gas holdup and shaft torque remained stable without systematic drift.

An air–water system was used as a standardized hydrodynamic reference for comparison of the original and redesigned agitation systems under identical fluid properties (Gimbun et al. 2009). This allowed the relative effects of the combined changes in impeller configuration and diameter on flow circulation, gas dispersion, mixing, and oxygen-transfer characteristics to be evaluated consistently. Because the viscosity, rheological behavior, biomass content, and interfacial properties of high-cell-density fermentation broth may influence gas–liquid hydrodynamics and mass transfer (Garcia-Ochoa and Gomez 2009), the CFD results were interpreted primarily as comparative predictions rather than absolute representations of the fermentation broth.

### Statistical analysis

For each fermentation condition, three independent fermentation batches were performed as biological replicates (*n* = 3). The independent fermentation batch was considered the experimental unit for statistical comparisons. Unless otherwise specified, experimental data were presented as mean ± standard deviation (SD). Prior to ANOVA, normality and homogeneity of variance were assessed using the Shapiro–Wilk test and Levene’s test, respectively. For replicated fermentation endpoints, statistical significance among experimental groups was evaluated using one-way analysis of variance (ANOVA) followed by Tukey’s post-hoc test, where applicable (*p* < 0.05). The RSM model fitting and significance were evaluated using ANOVA within the Design-Expert software.

## Results and discussion

### Construction of the *K. phaffii* strain

The xylanase‑coding gene *xynC* (GenBank: AJ278385.1) was codon‑optimized for *K. phaffii* and chemically synthesized by Sangon Biotech. Target gene fragments with homology arms to pPICZa were amplified by PCR. Vector pPICZa was double‑digested with *Eco*RI and *Not*I. The linearized vector was recovered after agarose‑gel separation. The *xynC* fragment was inserted into the vector via one‑step homologous recombination. The ligation mixture was transformed into *Escherichia coli* DH5α. Zeocin‑resistant transformants were screened by colony‑PCR and sequencing to yield recombinant plasmid *xynC*-pPICZa (Fig. S4). After *Sac*I linearization, *xynC*-pPICZa was electroporated into *K. phaffii* competent cells. Transformants were cultivated on Zeocin‑containing YPD plates, and single colonies were sub‑cultured for strain preservation. 

### Systematic optimization of $\:{\boldsymbol{k}}_{\boldsymbol{L}}\boldsymbol{a}$ via response surface methodology (RSM)

In high-cell-density aerobic fermentation cultures of *K. phaffii*, oxygen supply was a rate-limiting step for the production of target proteins due to the low solubility of oxygen in aqueous media (Liu et al. 2016). While enhancement of $\:{k}_{L}a$ (the volumetric oxygen mass transfer coefficient) was recognized as a key strategy to alleviate oxygen limitation (Oliveira et al. 2004), systematic investigation of how agitation speed, aeration rate, and headspace pressure jointly affected oxygen-transfer performance remained insufficient, particularly with the consideration of equipment tolerance and process economic constraints (Knoll et al. 2005). Therefore, this study employed the response surface methodology (RSM) to comprehensively evaluate the interactive effects of these operational parameters on $\:{k}_{L}a$ in a 5 L bioreactor. The experimental design and results were presented in Table 2.

A highly significant second-order polynomial model was fitted to the experimental data (F = 127.2, *P* < 0.0001). The lack of fit was not significant (*P* = 0.291). The model showed a high coefficient of determination (R^2^ = 0.9956), with an adjusted R^2^ of 0.9878 and a predicted R² of 0.9427. The adequate precision value was 30.73, well above the generally accepted value of 4, while the coefficient of variation (CV) was 3.938%, indicating good model precision and reproducibility (Table 4). The analysis of their main effects revealed a distinct hierarchy of their influence on $\:{k}_{L}a$: Agitation Speed (A) > Headspace Pressure (C) > Aeration Rate (B). This underscored the dominant role of agitation in generating interfacial area through bubble breakup, consistent with classical correlations, where $\:{k}_{L}a$ scaled with power input per unit volume (García-Ochoa and Gómez 2005; Moucha et al. 2012). Notably, headspace pressure emerged as the second most influential factor, surpassing aeration rate in its impact on $\:{k}_{L}a$ within the studied range of process parameters. These results identified headspace pressure as an important engineering factor influencing $\:{k}_{L}a$ within the investigated operating range.

**Table 4:** ANOVA for the fitted quadratic model of the$\:{k}_{L}a$

Source	Sum of squares	DF	Mean square	F-value	*P*-value
Model	28,570	9	3174	127.2	< 0.0001
A	6205	1	6205	248.5	< 0.0001
B	1758	1	1758	70.43	0.0003936
C	2298	1	2298	92.06	0.0002084
AB	107.1	1	107.1	4.291	0.09307
AC	1034	1	1034	41.4	0.001347
BC	759	1	759	30.4	0.002685
A^2^	7144	1	7144	286.2	< 0.0001
B^2^	5512	1	5512	220.8	< 0.0001
C^2^	6264	1	6264	250.9	< 0.0001
Residual	124.8	5	24.97	–	–
Lack of fit	99.25	3	33.08	2.587	0.291
Pure error	25.58	2	12.79	–	–
Cor total	28,700	14	–	–	–

Model statistics: R^2^ = 0.9956, adjusted R^2^ = 0.9878, predicted R^2^ = 0.9427, CV = 3.938%, and Adeq Precision = 30.73. A, agitation speed; B, aeration rate; C, headspace pressure

Furthermore, significant interaction effects were identified between Agitation and Pressure (AC, *P* < 0.01) and between Pressure and Aeration (BC, *P* < 0.01). To visualize these complex interactions, response surface plots were generated based on the model (see Fig. S5). The positive AC interaction was illustrated in Fig. S5b, where $\:{k}_{L}a$ increased sharply with rising pressure in the high agitation region, forming a distinct ridge. This suggested that the benefit of increased pressure on the driving force ($\:{C}^{*}-{C}_{l}$) was synergistically amplified under higher agitation, likely due to improved gas dispersion and interfacial renewal. Conversely, the negative BC interaction was evident in Fig. S5a, which showed that at high pressures, the surface flattened, indicating diminishing marginal gained in $\:{k}_{L}a$ from increased aeration. This may be attributed to changes in bubble coalescence behavior or the saturation of gas holdup under pressurized conditions (Lopes et al. 2013). Additionally, Fig. S5c further illustrated the combined response of $\:{k}_{L}a$ to agitation and aeration. These interactions, graphically validated by the response surfaces, underscored the necessity of multi-variable optimization over one-factor-at-a-time approaches (Liu et al. 2025). The final coded model equation was presented as Eq. (13):

13 $$ \begin{aligned} Y = \,& 192.90~ + ~27.85A~ + ~14.82B~ + ~16.95C~ \\ & + ~5.18AB~ + ~16.07AC - ~13.78BC~ \\ & - ~43.99A^{2} ~ - 38.64B^{2} ~ - ~41.19C^{2} \\ \end{aligned} $$

The stationary point of the fitted quadratic response surface was located at approximately 775 rpm agitation, 2.09 vvm aeration, and 1.12 bar headspace pressure, corresponding to a predicted $\:{k}_{L}a$ of approximately 201.5 h^− 1^. For the subsequent fermentation experiments, 700 rpm, 2.0 vvm, and 1.0 bar were selected as a practical operating condition within the experimentally evaluated region and the defined fermentation operating limits. At this selected operating condition, the model predicted a $\:{k}_{L}a$ of 192.9 h^− 1^, while independent validation experiments yielded 190.5 ± 3.2 h^− 1^, corresponding to a relative prediction error of approximately 1.2%. The RSM analysis therefore quantitatively characterized the engineering oxygen-transfer capacity represented by $\:{k}_{L}a$, whereas the subsequent fermentation experiments evaluated the corresponding process responses using DO, OUR, biomass accumulation, and xylanase production.

### Effects of headspace-pressure regulation on oxygen-transfer and fermentation performance

**Fig. 1:**
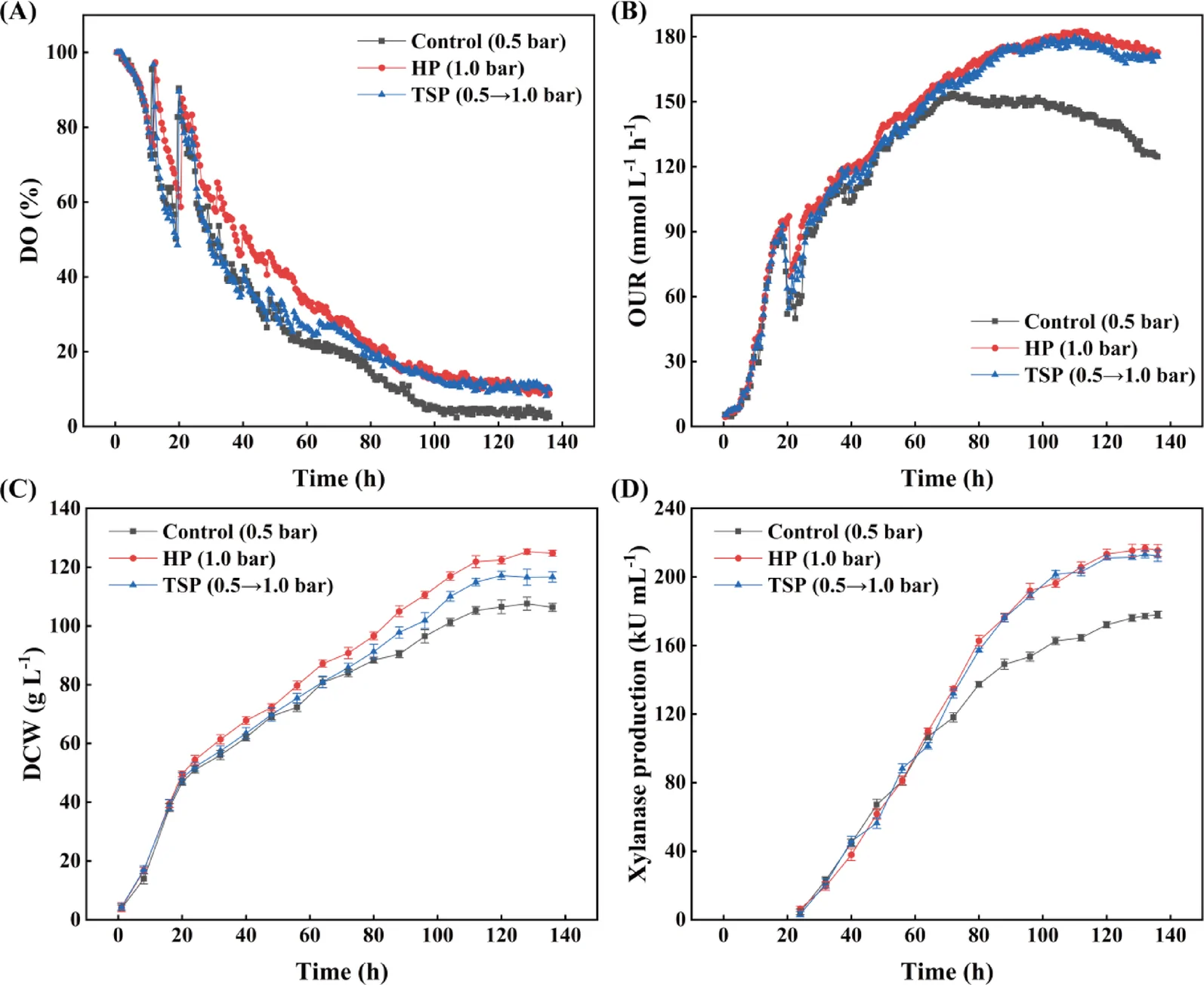
Dynamic profiles of key physiological and performance parameters of the fermenter under different headspace pressure control strategies. **A** Dissolved oxygen (DO) concentration. **B** Oxygen uptake rate (OUR). **C** Dry cell weight (DCW). **D** Xylanase production. DO and OUR profiles represent the mean values of three independent fermentation batches (*n* = 3), whereas DCW and xylanase activity are presented as mean ± SD (*n* = 3)

Because the three 5 L treatments were initiated under the same cultivation conditions and operated with identical feeding, agitation, and aeration profiles, the observed differences among the Control, HP, and TSP groups primarily reflected the different headspace-pressure profiles. The key process and fermentation-performance parameters are summarized in Table 5, with their time-course profiles shown in Fig. 1. DO and OUR were used to characterize the oxygen supply–demand state during fermentation, whereas DCW and xylanase activity represented biomass accumulation and product formation, respectively.

**Table 5:** Comparison of the key physiological parameters and fermentation performance under three different headspace pressure control strategies, i.e., control: constant conventional pressure; HP: constant high pressure; and TSP: two-stage dynamic pressure

Parameters	Control (0.5 bar)	HP (1.0 bar)	TSP (0.5→1.0 bar)
Specific growth rate,$\:\mu\:$(h^− 1^)	0.129 ± 0.010^a^	0.130 ± 0.011^a^	0.128 ± 0.011^a^
Dry cell weight (DCW, g/L)	108.15 ± 1.15^c^	125.25 ± 0.91^a^	120.25 ± 1.04^b^
Peak oxygen uptake rate (OUR, mmol L^− 1^ h^− 1^)	153.66 ± 4.90^b^	182.59 ± 3.31^a^	179.93 ± 3.78^a^
Apparent DO threshold ($\:{C}_{crit}$, % of saturation)	20.3 ± 2.1^a^	12.5 ± 1.0^b^	11.3 ± 1.5^b^
Maximum xylanase activity (KU/mL)	177.95 ± 2.04^b^	216.80 ± 2.06^a^	213.23 ± 2.10^a^
Acetate accumulation (g/L)	2.13 ± 0.26^a^	1.22 ± 0.13^b^	1.16 ± 0.15^b^
Overall volumetric xylanase productivity (KU/mL/h)	1.308 ± 0.015^b^	1.594 ± 0.015^a^	1.568 ± 0.015^a^
Overall biomass-specific productivity (KU/g_DCW_/h)	12.10 ± 0.19^b^	12.73 ± 0.18^a^	13.04 ± 0.27^a^

Values are expressed as mean ± standard deviation (SD) from three independent fermentation batches (*n* = 3). Different superscript lowercase letters within the same row indicate statistically significant differences among the three pressure-control strategies (one-way ANOVA followed by Tukey’s post-hoc test, *p* < 0.05)

In the Control group (0.5 bar), DO remained above 50% during the initial growth phases (Phases I and II). After entering the methanol-induction phase (Phase III), increasing oxygen demand associated with biomass accumulation resulted in a progressive decline in DO after approximately 72 h, eventually reaching below 20%. Concomitantly, OUR reached a peak of 153.66 ± 4.90 mmol/L/h and subsequently decreased to 124.5 mmol/L/h during the late induction stage, corresponding to an approximately 19.0% decline from the peak value. This decrease suggested that oxygen supply became insufficient to sustain the previous level of respiratory activity (Garcia-Ochoa et al. 2010; Yu et al. 2010). Even when agitation and aeration reached their maximum operational levels of 700 rpm and 2.0 vvm, respectively, DO could not be restored, indicating that agitation and aeration alone were insufficient to meet the increasing oxygen demand within the predefined operating limits under the conventional-pressure condition.

In contrast, both pressure-enhanced strategies improved oxygen availability during the induction phase. The HP group maintained a higher DO level, while OUR increased and remained at a higher level without the subsequent decline observed in the Control group. The TSP group initially followed DO and OUR profiles similar to those of the Control group at 0.5 bar. Following the stepwise increase in headspace pressure from 0.5 to 0.75 and subsequently to 1.0 bar during the mid-induction phase, DO rapidly recovered and stabilized, while the OUR profile approached that observed in the HP group (Fig. 1A, B). Because identical agitation and aeration profiles were used for all three treatments, the recovery of DO and maintenance of higher OUR following pressure elevation were consistent with the expected increase in the oxygen-transfer driving force.

Although the specific growth rates were comparable among the three groups (~ 0.13 h^− 1^), clear quantitative differences were observed in OUR, biomass accumulation, and xylanase production. Peak OUR values reached 182.59 ± 3.31 and 179.93 ± 3.78 mmol/L/h in the HP and TSP groups, respectively, corresponding to increases of 18.8% and 17.1% relative to the Control group. Final DCW values reached 125.25 ± 0.91 and 120.25 ± 1.04 g/L, representing increases of 15.8% and 11.2%, respectively, compared with the Control group (108.15 ± 1.15 g/L). The apparent DO thresholds estimated from the fermentation profiles were approximately 12.5 ± 1.0% for the HP group and 11.3 ± 1.5% for the TSP group, compared with 20.3 ± 2.1% for the Control group. Thus, higher OUR values were maintained over a lower observed DO range under elevated-pressure conditions. Maximum xylanase activities reached 216.80 ± 2.06 and 213.23 ± 2.10 KU/mL in the HP and TSP groups, respectively, corresponding to increases of 21.8% and 19.8% relative to the Control group (177.95 ± 2.04 KU/mL). Overall, the approximately 17–19% increase in peak OUR under the pressure-enhanced conditions was accompanied by an approximately 11–16% increase in final biomass and a 20–22% increase in maximum xylanase activity, showing a consistent quantitative association between maintained respiratory activity and fermentation performance. This trend is consistent with previous reports showing that enhanced oxygen transfer through pressure elevation can improve heterologous protein production in *K. phaffii* (Knoll et al. 2005; Lopes et al. 2014).

The TSP condition also exhibited the lowest acetate accumulation among the three pressure strategies. Acetate accumulation was 1.16 g/L, representing a 45.5% reduction relative to the Control group. Together with the higher xylanase activity observed under TSP, the reduced acetate accumulation further supported the favorable fermentation performance of the dynamic-pressure strategy. Statistical analysis showed that both HP and TSP significantly increased peak OUR, DCW, maximum xylanase activity, and overall xylanase productivities compared with the Control group (*p* < 0.05, Table 5). Both pressure-enhanced strategies also significantly reduced the apparent DO threshold and acetate accumulation (*p* < 0.05). No significant differences were observed between HP and TSP in peak OUR, maximum xylanase activity, acetate accumulation, or overall xylanase productivities (*p* > 0.05), whereas DCW was significantly higher under HP (*p* < 0.05). Specific growth rates did not differ significantly among the three groups (*p* > 0.05). Compared with constant high pressure, the TSP strategy maintained conventional pressure during the early stage and applied elevated pressure only during the later induction stage as oxygen demand increased. Consequently, TSP shortened the duration of elevated-pressure operation while maintaining favorable oxygen-transfer and fermentation performance. However, elevated-pressure operation requires pressure-rated equipment and reliable pressure-control systems and may increase gas-compression requirements and associated operating costs. Increased pressure may also affect CO_2_ stripping and dissolved CO_2_ accumulation, with potential strain- and process-dependent effects on cell physiology. These engineering and physiological constraints should therefore be considered together with the oxygen-transfer benefits when applying pressure-based strategies at larger scales.

### Scale-up verification from 5 L to 1000 L pilot scale

**Fig. 2:**
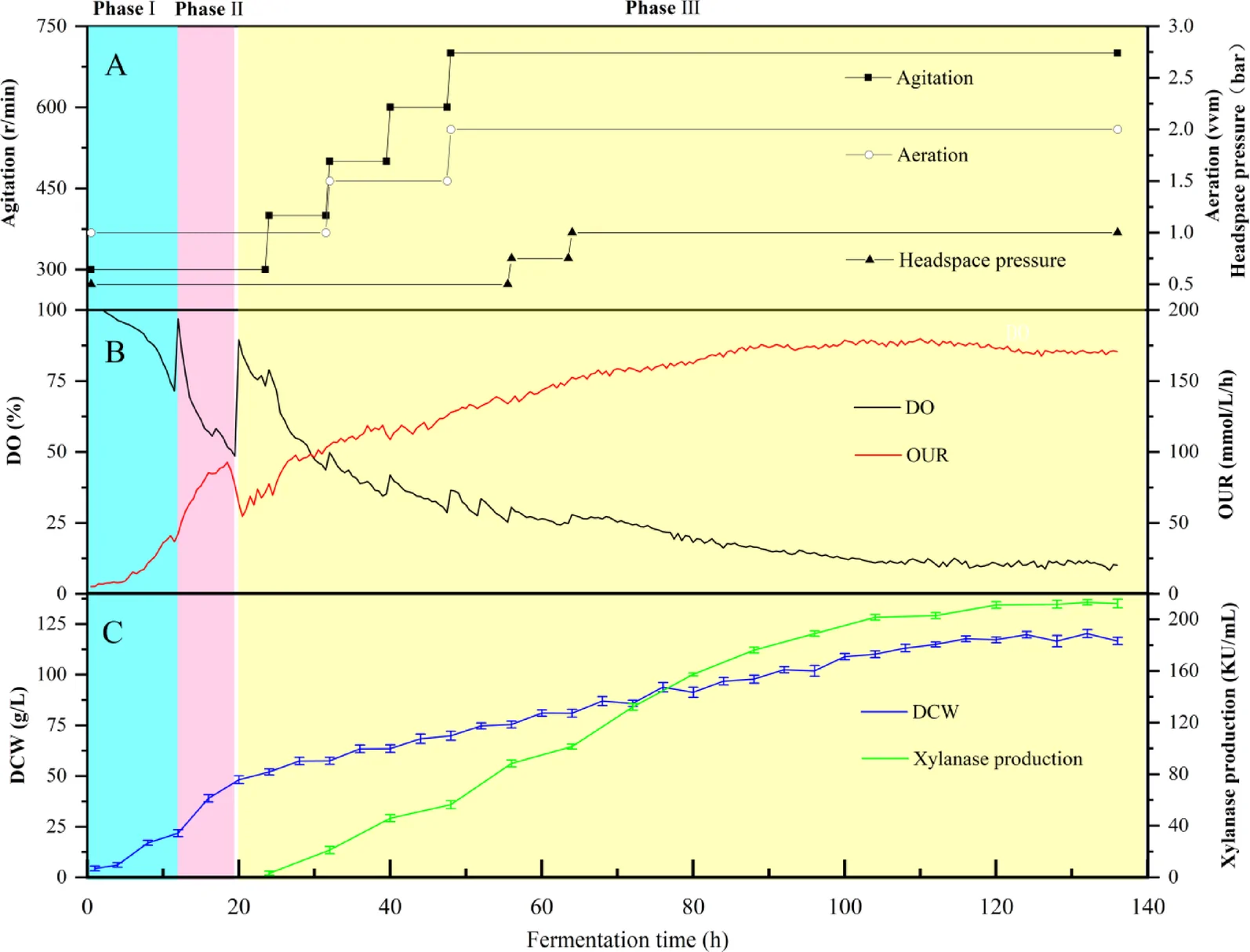
Baseline integrated fermentation profiles of the 5 L fermenter upon the physiology-based two-stage dynamic pressure (TSP) control strategy. **A** Fermenter operation, **B** Physiology (DO, OUR), and **C** Performance (DCW, Xylanase production). DO and OUR profiles represent the mean values of three independent fermentation batches (*n* = 3), whereas DCW and xylanase activity are presented as mean ± SD (*n* = 3)

**Fig. 3:**
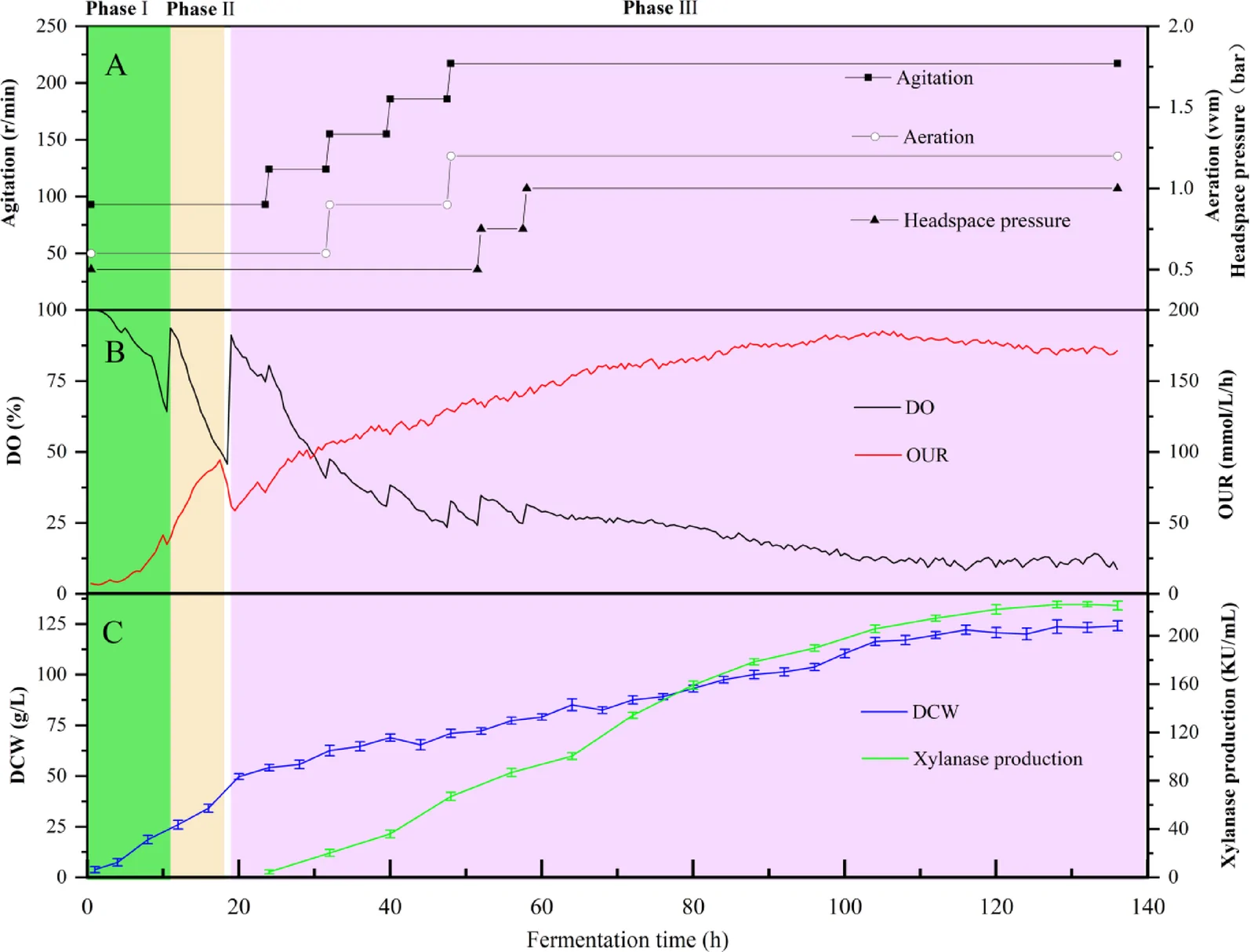
Scale-up of the bioreactor from 5 L to 1000 L: maintenance of the key physiological and performance profiles in the three phases of the fermenter operation. **A** Fermenter operation, **B** Physiology (DO, OUR), and **C** Performance (DCW, Xylanase production). DO and OUR profiles represent the mean values of three independent fermentation batches (*n* = 3), whereas DCW and xylanase activity are presented as mean ± SD (*n* = 3)

The TSP-based control strategy was further implemented in the 1000 L pilot-scale fermenter. The OUR profiles at the 5 L and 1000 L scales showed similar overall temporal patterns, characterized by a progressive increase in oxygen demand during biomass accumulation and sustained high OUR during the methanol-induction phase (Fig. 2 and Fig. 3). Quantitatively, the peak OUR was 179.93 mmol/L/h at 5 L and 185.23 mmol/L/h at 1000 L, corresponding to a difference of approximately 2.9% between the two scales.

Fermentation-performance indicators were also maintained within a comparable range after scale-up. At 136 h, the final DCW was 120.25 ± 1.04 g/L at 5 L and 124.08 ± 2.47 g/L at 1000 L. The corresponding xylanase activities were 213.23 ± 2.10 and 225.07 ± 3.64 KU/mL, respectively. Because both fermentations were completed at 136 h, the overall volumetric xylanase productivities were 1.568 and 1.655 KU/mL/h, while the corresponding overall biomass-specific productivities were 13.04 and 13.34 KU/g_DCW_/h at the 5 L and 1000 L scales, respectively.

The 1000 L agitation conditions corresponded to a calculated specific-power ratio of approximately 1.05 relative to the 5 L scale, while aeration was adjusted within the feasible operating range of the pilot-scale fermenter. Although the detailed DO trajectories differed between the two reactors, similar OUR patterns and comparable biomass accumulation, xylanase production, and overall productivity were obtained at the two scales. Thus, the main fermentation characteristics observed at the 5 L scale were largely retained after scale-up to 1000 L.

### CFD-guided agitation-system redesign at the 30 m^3^ scale

During operation of the original 30 m^3^ fermenter equipped with four Rushton turbines, oxygen-transfer limitation became evident during the high-oxygen-demand methanol-induction stage despite implementation of the TSP strategy. Across three independent fermentation batches, DO decreased below 25% at approximately 54–62 h even after the headspace pressure had been increased to 1.0 bar, and subsequently declined to below 10% during the later induction stage. This decrease was accompanied by an earlier decline in OUR. The highest peak OUR observed across the three batches was 145.62 mmol/L/h. At 136 h, the final DCW and xylanase activity were 116.53 ± 3.09 g/L and 198.76 ± 2.45 KU/mL, respectively. These observations indicated that pressure-based process regulation alone was insufficient to maintain oxygen availability and fermentation performance in the original 30 m^3^ configuration, motivating further investigation of reactor-scale hydrodynamic limitations.

CFD simulation was therefore employed to examine the multiphase flow field and gas–liquid mass-transfer characteristics of the 30 m^3^ fermenter and to characterize potential hydrodynamic limitations associated with the original four-stage Rushton turbine (RT) agitation system. The CFD results showed that the radial-flow-dominated pattern of the original RT system resulted in pronounced compartmentalization of the flow field and insufficient axial circulation, thereby limiting large-scale mixing and gas dispersion (Vrábel et al. 2000; Gu et al. 2026). The CFD-predicted average $\:{k}_{L}a$ of the original configuration was 121.6 h^− 1^. Together with the predicted flow-field characteristics, this result indicated that reactor-scale hydrodynamics could constrain oxygen-transfer performance at the 30 m^3^ scale and motivated further optimization of the agitation system.

**Fig. 4:**
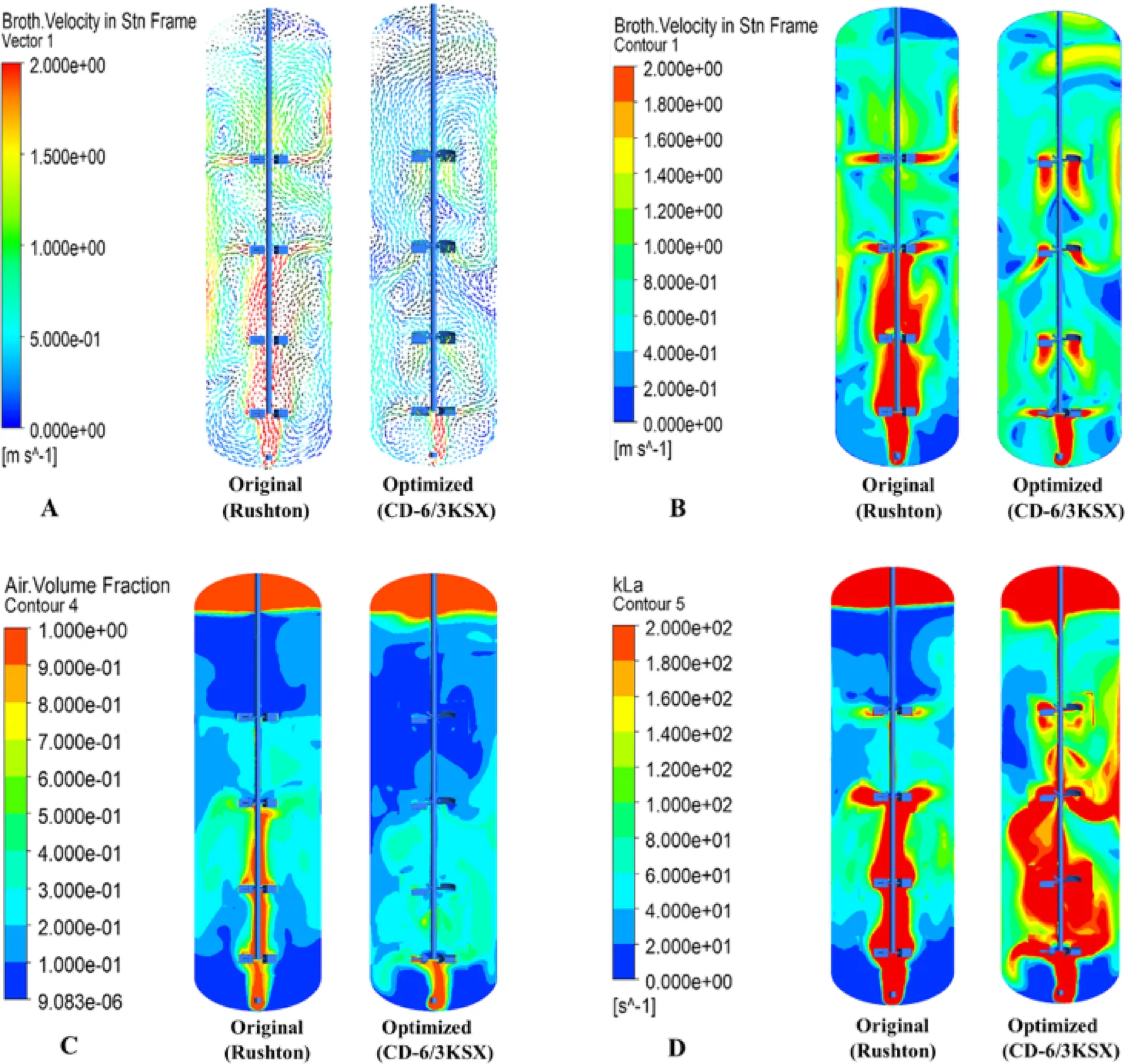
CFD-predicted flow and mass-transfer characteristics of the original and redesigned agitation systems in the 30 m^3^ bioreactor. **A** Velocity vector plot, **B** Velocity contour, **C** Gas holdup distribution, and **D** Volumetric oxygen transfer coefficient ($\:{k}_{L}a$) distribution

The agitation system was subsequently redesigned by jointly modifying the impeller configuration and impeller diameter. The bottom RT impeller was replaced with a CD-6 radial-flow impeller to improve gas dispersion and initial bubble breakup (Gimbun et al. 2009), while the three upper RT impellers were replaced with 3KSX axial-flow impellers to strengthen axial circulation and improve mixing homogeneity (Gogate et al. 2000; Vrábel et al. 2000). The impeller diameter was also increased from 0.7 to 0.8 m to enhance pumping capacity and gas–liquid contacting. CFD simulation of the redesigned system predicted stronger global circulation and reduced low-velocity regions (Fig. 4). Under identical simulated operating conditions, the combined redesign increased the CFD-predicted average $\:{k}_{L}a$ from 121.6 to 178.0 h^− 1^, corresponding to a 46.4% increase, while the predicted mixing time decreased from 41.8 to 23.7 s, corresponding to a 43.3% reduction. These CFD results provide a comparative assessment of the hydrodynamic performance of the two agitation configurations under identical simulated conditions. Overall, the redesigned agitation system showed improved predicted circulation, mixing, and oxygen-transfer characteristics.

**Fig. 5:**
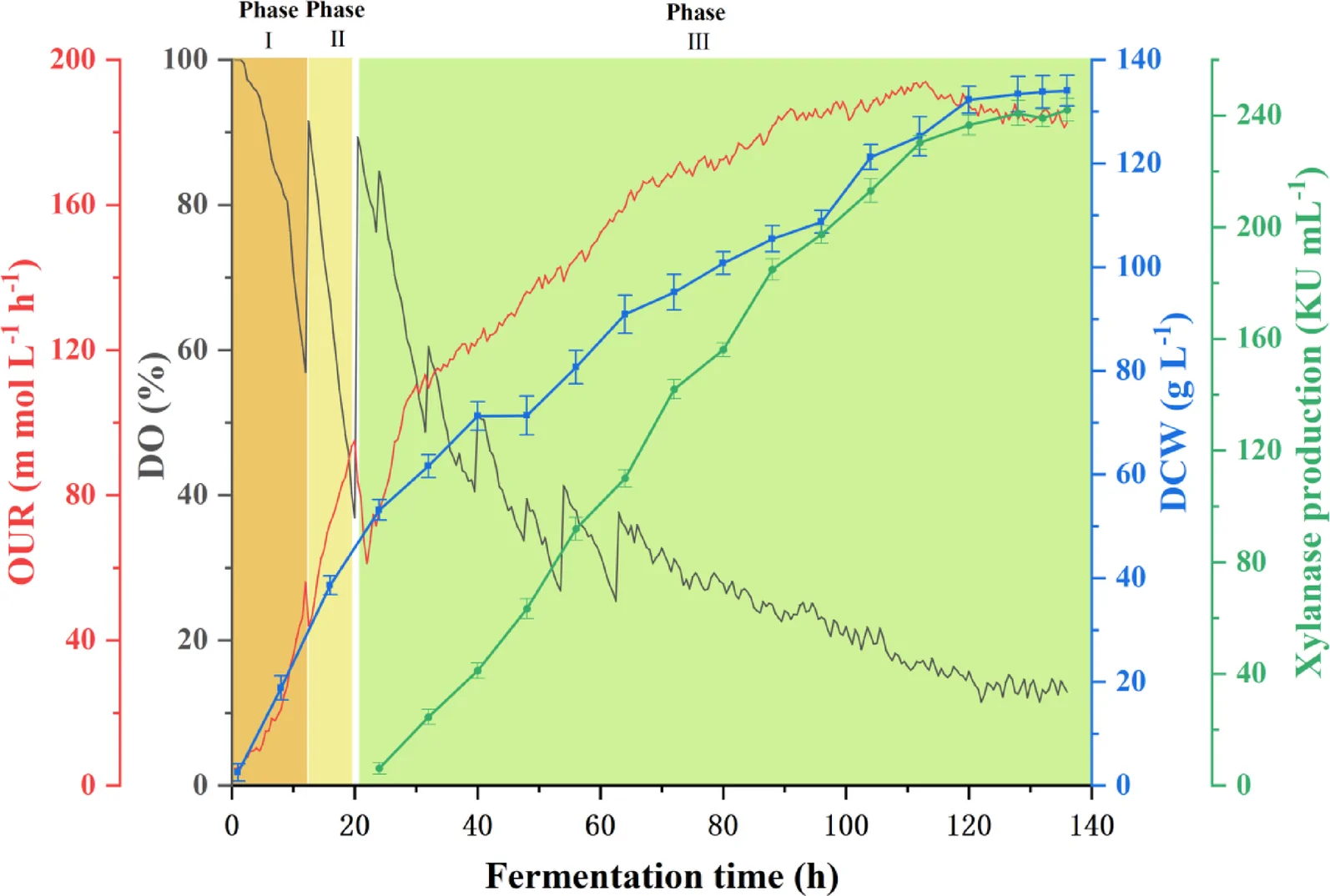
Dynamic profiles of the key physiological and performance parameters in the 30 m^3^ industrial-scale fermentation process with the optimized agitation system. DO and OUR profiles represent the mean values of three independent fermentation batches (*n* = 3), whereas DCW and xylanase activity are presented as mean ± SD (*n* = 3)

Following implementation of the CFD-guided agitation-system redesign, fermentation was conducted in the retrofitted 30 m^3^ production vessel using the TSP operating strategy. During the mid-to-late methanol-induction phase, DO remained stable, OUR was maintained at a high level, and both biomass concentration and xylanase activity continued to increase (Fig. 5). At 136 h, the final DCW reached 134.01 ± 2.98 g/L, while the final xylanase activity reached 242.02 ± 4.07 KU/mL. Compared with the original Rushton-turbine configuration operated with the TSP strategy, the redesigned system increased the final DCW and xylanase activity by approximately 15.0% and 21.8%, respectively. The corresponding overall volumetric xylanase productivity was 1.780 KU/mL/h, and the overall biomass-specific productivity was 13.28 KU/g_DCW_/h. For comparison, the corresponding volumetric productivities were 1.568 and 1.655 KU/mL/h at the 5 L and 1000 L scales, respectively, while the biomass-specific productivities were 13.04 and 13.34 KU/g_DCW_/h. The biomass-specific productivities were therefore similar across the three scales, whereas the overall volumetric productivity increased from 1.568 KU/mL/h at 5 L to 1.780 KU/mL/h at 30 m^3^.

## Conclusions

This study developed a multiscale oxygen-transfer enhancement approach integrating dynamic pressure regulation with CFD-guided reactor optimization for high-density *K. phaffii* fermentation. At the 5 L scale, pressure-enhanced operation improved respiratory activity, biomass accumulation, and xylanase production, and the TSP strategy was subsequently implemented at the 1000 L scale with comparable oxygen-demand and fermentation-performance characteristics. At 136 h, the final DCW and xylanase activity were 120.25 ± 1.04 g/L and 213.23 ± 2.10 KU/mL at 5 L, 124.08 ± 2.47 g/L and 225.07 ± 3.64 KU/mL at 1000 L, and 134.01 ± 2.98 g/L and 242.02 ± 4.07 KU/mL at 30 m^3^, respectively. At the industrial scale, CFD-guided redesign of the agitation system resulted in a predicted 46.4% increase in average $\:{k}_{L}a$ and a predicted 43.3% reduction in mixing time under identical simulation conditions. Overall, these results demonstrate the value of combining process-level oxygen-transfer regulation with reactor-level hydrodynamic optimization for the recombinant *K. phaffii* process investigated here, while the applicability of this strategy to other strains and bioreactor configurations requires further evaluation.

## Supplementary Information

Below is the link to the electronic supplementary material.

Supplementary Material 1.

## Data Availability

The dataset(s) supporting the conclusions of this article are included within the article and its additional file(s). Additional raw data are available from the corresponding author upon reasonable request.
