# Supplementary material for: Multiscale oxygen transfer enhancement in high‑density Komagataella phaffii fermentation: dynamic pressure control and CFD‑guided industrial scale‑up
Source: Bioresour Bioprocess. 2026 Oct 28;13(1):158. doi: 10.1186/s40643-026-01155-9 (PMC13713958; doi:10.1186/s40643-026-01155-9)
Supplement: Supplementary file 1 [file 40643_2026_1155_MOESM1_ESM.docx]

**Multiscale oxygen transfer enhancement in high‑density *Komagataella phafﬁi* fermentation: dynamic pressure control and CFD‑guided industrial scale‑up**

Qiang Duan^1,2,3,4^, Fengchao Wang ^4^, Qinran Ding^1,2,3^, Bo Xiong^1,2,3^, Baocai Zhang^5^, Huan Yu^5^, Junqi Zhang^5^, Yaru Chen^5^, Chao Li^5^, Qitian Huang^5^, Hao Song^1,2,3,5^*

^1^ State Key Laboratory of Synthetic Biology, Tianjin University, Tianjin 300350, China

^2^ Frontiers Science Center for Synthetic Biology (Ministry of Education), Tianjin University, Tianjin 300350, China

^3^ School of Synthetic Biology and Biomanufacturing, Tianjin University, Tianjin 300350, China

^4^ Weifang KDN Biotech Co., Ltd., Weifang, Shandong 261500, China

^5^ Institute of Synthetic Biology, College of Life and Health Sciences, Northeastern University, Shenyang, 110169, China

* Corresponding author: H.S.

Email: [hsong@tju.edu.cn](mailto:hsong@tju.edu.cn)

**Figure S1.** Calibration relationship between equivalent OD600 and gravimetrically determined dry cell weight (DCW) for the recombinant *K. phaffii* strain.

**Figure S2.** Schematic diagram of the structure of the 30 m³ fermenter. (a) before optimization; (b) after optimization.

**Mesh independence study**

To evaluate the influence of mesh resolution on the CFD predictions, a mesh independence study was performed using four mesh configurations containing approximately 0.43, 0.85, 1.25, and 2.17 million cells, respectively. The average liquid velocity, average gas velocity, average turbulent energy dissipation (TED), and average gas holdup were selected as representative global hydrodynamic parameters for assessing mesh convergence.

As shown in Fig. S3, these parameters changed noticeably as the mesh number increased from 0.43 to 1.25 million cells. However, further refinement from 1.25 to 2.17 million cells resulted in only minor variations in all four monitored parameters, indicating that the numerical solution had approached mesh independence. Considering the balance between numerical accuracy and computational cost, a mesh resolution of approximately 1.25 million cells was selected as the reference mesh density. The same meshing strategy and local-refinement criteria were subsequently applied to the redesigned configuration, resulting in a final mesh of approximately 1.30 million cells.

**Figure S3.** Mesh independence study for the bioreactor CFD model. (A) Average liquid velocity; (B) average gas velocity; (C) average turbulent energy dissipation (TED); and (D) average gas holdup at different mesh resolutions.

**Figure S4.** The *xynC*-pPICZa plasmid map. To construct the plasmid, two primers *xynC*-U-Za-F and *xynC*-U-Za-R were used:

*xynC*-U-Za-F: GAAAAGAGAGGCTGAAGCTTTTCCTTCTGAGTTGGCTCAAAG
*xynC*-U-Za-R: CTAGAAAGCTGGCGGCCGCTTAAGAGACGGTAATAGTAG

**Figure S5.** Response surfaces of $k_{L}a$ as a function of (a) aeration rate and headspace pressure, (b) agitation speed and headspace pressure, and (c) agitation speed and aeration rate.
